# Rationale and design of two prospective, multicenter, observational studies on reproductive outcome in women with recurrent failures after spontaneous or assisted conception: OTTILIA and FIRST registries

**DOI:** 10.1186/s12884-019-2444-y

**Published:** 2019-08-13

**Authors:** Michela Villani, Domenico Baldini, Pasquale Totaro, Giovanni Larciprete, Mirjana Kovac, Domenico Carone, Serena Maria Passamonti, Eleonora Tamborini Permunian, Tiziana Bartolotti, Andrea Lojacono, Rossella Cacciola, Giuliano Lo Pinto, Eugenio Bucherini, Valerio De Stefano, Corrado Lodigiani, Cristina Lavopa, Yoon Sung Cho, Caterina Pizzicaroli, Donatella Colaizzo, Elvira Grandone

**Affiliations:** 1 Thrombosis and Haemostasis Unit, I.R.C.C.S. “Casa Sollievo della Sofferenza” Poliambulatorio Giovanni Paolo II, Viale Padre Pio, San Giovanni Rotondo, Italy; 2 Centro di Fecondazione Medicalmente Assistita MoMò Fertilife, Bisceglie, Italy; 3 Centro PMA “Casa di Cura Santa Maria”, Bari, Italy; 4 Department of Obstetrics and Gynecology, Fatebenefratelli Isola Tiberina Hospital, Rome, Italy; 5 Blood Transfusion Institute of Serbia, Belgrade, Serbia; 6 Center of Reproduction and Andrology (CREA), Taranto, Italy; 7 A. Bianchi Bonomi Hemophilia and Thrombosis Center, Fondazione IRCCS Ca’ Granda - Ospedale Maggiore Policlinico, Milan, Italy; 8 Department of Clinical Medicine, Insubria University, Varese, Italy; 9 Centro PMA “ARTeBIOS”, Lugo, Ravenna, Italy; 10 Obstetrics and Gynecology, Department of Clinical and Experimental Sciences, ASST Spedali Civili and University of Brescia, Brescia, Italy; 11 Haemostasis Unit, Department of Clinical and Experimental Medicine, University of Catania, Catania, Italy; 12 Department of Internal Medicine, Galliera Hospital, Genoa, Italy; 13 Unit of Vascular Medicine and Angiology, Civic Hospital of Faenza, Faenza, Italy; 14 Institute of Hematology, Catholic University, Rome, Italy; 15 Thrombosis and Hemorrhagic Center, Humanitas Research Hospital and Humanitas University, Rozzano, Italy; 16 Ob/Gyn Department of the First I.M. Sechenov Moscow State Medical University, Moscow, Russian Federation

**Keywords:** Pregnancy loss, Assisted reproductive technologies, Implantation failure, Observational study, Thrombophilia, Risk factors, Heparins

## Abstract

**Background:**

Spontaneous pregnancy loss and implantation failure after assisted reproductive technologies (ART) are very common occurrences. Although 50–60% of all cases remains unexplained, various predisposing factors, including thrombophilias, have been identified. Thus, the potential benefit of a prophylaxis with low-molecular-weight heparins in improving outcomes has been often investigated over the years. However, the majority of studies are observational and results from randomized clinical trials (RCTs) are inconclusive, probably due to heterogeneity and limited sample size. To cover these unmet needs and to have further data mainly based on the real-life clinical management, we designed these multicenter registries.

**Methods:**

OTTILIA (Observational sTudy on antiThrombotic prevention in thrombophILIA and pregnancy loss) and FIRST (recurrent Failures in assIsted Reproductive Techniques) registries are two prospective, multicenter, observational studies to evaluate pregnancy or ART outcomes in consecutive women with previous reproductive failures after spontaneous or assisted conception, respectively. All enrolled women are observed from their first visit after positive pregnancy test (OTTILIA) or before commencing a new ART cycle (FIRST) until the end of pregnancy or ART procedure (negative pregnancy test/end of pregnancy, if successful cycle), respectively. Data are collected by means of questionnaires and recorded in a central database. Follow-up investigations are performed during hospital stay, routine clinical follow-up visits or telephone interviews. Primary outcome is live birth rate in the OTTILIA register and clinical pregnancy rate in the FIRST.

**Discussion:**

Although RCTs are the ‘gold standard’ for evaluating treatment outcomes, we believe that our registries represent a valid alternative in improving knowledge on mechanisms involved in reproductive failures and supporting future clinical decisions.

**Trial registration:**

NCT 02385461, retrospectively registered 5 March 2015 (OTTILIA); NCT 02685800, registered 10 February 2016 (FIRST).

## Background

Spontaneous pregnancy loss (PL) and implantation failure after assisted reproductive technologies (ART) represent very common occurrences in women’s reproductive life.

It has been estimated that PL (generally defined as a spontaneous loss of pregnancy from conception to 20 weeks of gestation) affects up to 1000–1500/10000 pregnancies/year (10–15% of pregnancies) and about 1% of all cases is a recurrent PL (RPL) [1]. It is also known that the risk of PL increases with increasing numbers of pregnancies, regardless of previous obstetric outcomes [2]. The risk is expected to be particularly relevant when all previous pregnancies resulted in PL: the likelihood of carrying subsequent pregnancies to term is about 80% after 2 PL, compared with 55–75% after 3 PL [2].

Poor outcomes have been reported in the field of ART as well. Based on the last report by the European Society of Human Reproduction and Embryology (ESHRE), in Europe, during 2012, the mean pregnancy rate per embryo transfer was 33.8% after in vitro fertilization (IVF), 32.3% after intracytoplasmic sperm injection (ICSI), 23.1% after frozen embryo replacement and 48.8% after egg donation [3].

Although various predisposing factors have been identified in both conditions, 50–60% of all cases remain unexplained. A potential causative role of inherited (the Factor V Leiden or the prothrombin A20210 gene variants, protein C, protein S or antithrombin deficiency) or acquired (antiphospholipid-antibody syndrome) thrombophilias has been documented [4–6], presumably due to the obstruction in placental vessels caused by haemostatic disorders. Thus, the potential benefit of a prophylaxis with low-molecular-weight heparins (LMWHs) in improving the live birth rate or outcomes in ART procedures has been often investigated and demonstrated by several studies, that are mostly limited by their observational nature or the limited sample size [7–12]. On the contrary, randomized clinical trials (RCTs) on this issue have shown inconclusive results, probably due to heterogeneity and, sometimes, small number of women included [13–18]. Therefore, despite increasing knowledge on reproductive failures over the past decades, there are still several aspects of clinical management, treatment and prognosis with uncertainties that need to be addressed. As far as ART, the need for further well-designed RCTs with larger sample size has also been raised by the last Cochrane review [19]. Unfortunately, RCTs in pregnant/infertile women are often stopped before completing, due to poor and slow recruitment.

To cover these unmet needs and to have further data mainly based on the real-life clinical management, we are currently conducting the OTTILIA (Observational sTudy on antiThrombotic prevention in thrombophILIA and pregnancy loss – NCT 02385461) and FIRST (recurrent Failures in assIsted Reproductive Techniques – NCT 02685800) registries, respectively.

We summarize here the design of these studies and discuss the importance of their results in the clinical practice.

## Methods

### Aims

Primary aims of the OTTILIA and FIRST registries are: (1) to identify and evaluate all possible factors associated with reproductive outcome and improve risk stratification of women, (2) to assess the best clinical management strategies (including a potential benefit of prophylaxis with LMWH) in improving outcome, (3) to evaluate characteristics of women who possibly benefit from an antithrombotic treatment.

Secondary aims are: (1) to evaluate occurrence of ovarian hyperstimulation syndrome (OHSS) in women undergoing ovarian stimulation, obstetric and thromboembolic complications according to characteristics of women and possible treatment, (2) to evaluate, among women prescribed with pharmacological treatments, the risk of side effects.

Additional aims include evaluation of incidence, prevalence, and recurrence of reproductive failures in the ‘real word’ of women trying to get pregnant and/or to carry a pregnancy to term.

### Design and setting of the study

OTTILIA and FIRST registries are two prospective, studies designed to investigate pregnancy or ART outcomes according to different clinical and laboratory features and clinical management in women consecutively observed because of previous reproductive failures after spontaneous or assisted conception, respectively.

The possible assignment of women to treatments (such as LMWH) is not decided in advance but instead falls within current practice.

Obstetrics and Gynecology Departments as well as Thrombosis Centers with local Ethical Committee approval are allowed to participate to these studies.

Authors adhered to SPIRIT guidelines.

### Participants

All consecutive women who meet the inclusion criteria for the OTTILIA or FIRST registries (Table 1) are recruited by participating Centers on admission after obtaining informed consent. Women eligible for the OTTILIA register can undergo thrombophilia test before conception, according to local policy. If the test is not available or the test was not prescribed by the physician, women included are considered as non- carriers. As far as FIRST, knowledge of thrombophilia status is not a prerogative of the register. However, when information is available, it will be considered in final analysis.

**Table 1:** Inclusion and exclusion criteria of the OTTILIA and FIRST registries

OTTILIA	FIRST
Inclusion criteria	
• Pregnancy confirmed by urine/blood pregnancy test or ultrasound examination • Previous recurrent otherwise unexplained PL * ≥3 or 2 PL in the presence of at least 1 normal fetal karyotype* or • At least 1 previous IUFD * A loss after 20 weeks of a morphologically normal fetus*	• ART cycle at enrolment *First, second and third level ART cycles* • 2 or more implantation failures/losses of clinical pregnancies after ART (independently of the number of embryos transferred) *At least 2 previous cycles with negative pregnancy test and/or PL after a positive pregnancy test*
Exclusion criteria	
• Personal history of venous and/or arterial thromboembolism • Allergy to LMWHs • Uterine abnormalities • Cervical incompetence • Chromosomal abnormalities in parents • Known hemorrhagic diathesis	• Uterine abnormalities • Hydrosalpinx • Chromosomal abnormalities in parents • Known hemorrhagic diathesis • Previous inclusion in the study

*PL* pregnancy loss, *IUFD* intrauterine fetal death, *ART* assisted reproductive technologies, *LMWHs* low-molecular-weight heparins

Enrolled women are free to withdraw their informed consent and exit from the study.

### Study procedure

All enrolled women are observed from their first visit after positive pregnancy test (OTTILIA) or before commencing a new ART cycle (FIRST) until the end of pregnancy or ART procedure (negative pregnancy test/end of pregnancy, if successful cycle), respectively.

At baseline, clinical information and index- pregnancy/ART cycle details are obtained from all women by a specially trained staff (Table 2). Data are collected by means of questionnaires and recorded in a web-based database including an electronic form for each woman enrolled. To ensure high quality data, a centralized data management performs quality control and plausibility checks according to predefined procedures. Follow-up data (Table 2) are collected during the three trimesters of pregnancy and in the puerperium period (6 weeks after delivery), at the time of hospital stay, routine clinical follow-up visits or telephone interviews.

**Table 2:** Baseline and Follow-up data for evaluation and monitoring of women enrolled in the OTTILIA and FIRST registries

OTTILIA	
Baseline Clinical information: • Maternal age • Body Mass Index • Smoking habit • Reproductive history (previous pregnancies and obstetric complications occurred) • Personal and family history of VTE • Presence of inherited or acquired thrombophilias • Thrombophilic risk factors Index-pregnancy information: • Results of performed imaging tests, (ultrasonography, Doppler ultrasonography) • Invasive prenatal testing (i.e. villous sampling, amniocentesis) • Antithrombotic prophylaxis (brand, doses/day, duration)	Follow-up Pregnancy outcomes: • Live birth (gestational age, delivery mode, sex, birth weight, fetal or maternal hemorrhage) • Obstetric complications (PL, IUFD, gestational hypertension, preeclampsia, HELLP syndrome, SGA newborns, placental abruption) • VTE
FIRST	
Baseline Clinical information: • Maternal age • Body Mass Index • Smoking habit • Reproductive history (previous spontaneous pregnancies, ART attempts) • Cause of infertility (female, male, mixed or idiophatic), • Personal and family history of VTE • Presence of inherited or acquired thrombophilias • Thrombophilic risk factors Index-ART cycle information: • Fertilization (autologous/heterologous) • Type of ART procedure (IUI, IVF, ICSI, Other) • Ovarian stimulation (brand, doses/day, duration) • Antithrombotic prophylaxis (brand, doses/day, duration), • Number of oocytes retrieved •Number of embryo transfers	Follow-up ART outcomes: • Clinical pregnancy • Live birth (gestational age, delivery mode, sex, birth weight, fetal or maternal hemorrhage) • Obstetric complications (PL, IUFD, gestational hypertension, preeclampsia, HELLP syndrome, SGA newborns, placental abruption) • OHSS (time of occurrence after ovarian stimulation, severity, estradiol levels pg/ml, treatment) • VTE

*VTE* venous thromboembolism, *IUI* intrauterine insemination, *IVF* in vitro fertilization, *ICSI* intracytoplasmic sperm injection, *PL* pregnancy loss, *IUFD* intrauterine fetal death, *SGA* small-for-gestational age, *OHSS ovarian hyperstimulation syndrome*

The primary outcome is the live birth rate in the OTTILIA register and live birth or clinical pregnancy rate in the FIRST. The definition of all primary and secondary outcomes is provided in Table 3.

**Table 3:** Definitions of primary and secondary outcomes in the OTTILIA and FIRST registries

	Definitions	Source
Clinical pregnancy	A pregnancy confirmed by ultrasonographic visualization of gestational sac or fetal heartbeat	Wang X et al. Fertil Steril 2003; 79: 577–9.
Live birth	The delivery of a live newborn	https://www.gfmer.ch/Medical_education_En/Live_birth_definition.htm
Pregnancy loss (PL)	A loss occurring ≤20 weeks	Regan L. BMJ 1991; 302: 543–4
Intrauterine fetal death (IUFD)	A loss occurring after 20 weeks	Royal College of Obstetricians & Gynaecologists. Late intrauterine fetal death and stillbrith (Green-top Guideline 55). London: Royal College of Obstetricians & Gynaecologists; 2010 [cited 2019 May 30]
Preterm birth	The delivery of an infant before completion of 37 weeks gestation	ACOG Committee Opinion No 579: definition of term pregnancy. Obstet Gynecol. 2013;122: 1139–40
Gestational hypertension (GH)	Blood pressure ≥ 140/90 mmHg after 20 weeks of gestation in a previously normotensive woman	www.nice.org.uk/guidance/cg107
Preeclampsia	Blood pressure ≥ 140/90 mmHg and proteinuria of 0.3 g or more in a 24-h urine specimen occurring after 20 weeks of gestation in a previous normotensive woman	www.nice.org.uk/guidance/cg107
HELLP syndrome	Thrombocytopenia (i.e. platelet count < 150,000 cells/μL), elevated AST and/or ALT (> 40 IU/L), increased LDH (> 600 IU/L), and hemolysis (increased LDH level)	Martin JN et al. Am J Obstet Gynecol 1999; 180: 1373–84.
Small-for-gestational age (SGA) newborns	Apparently healthy neonate with birthweight <10th centile for gestational age in the absence of infection and maternal drug or alcohol abuse	RCOG. The investigation and management of the small–for–gestational–age fetus. Green-top Guideline. 2nd ed.; 2014
Placental abruption	The early separation of the placenta from the uterus	Ananth CV, et al. Am J Obstet Gynecol. 2016;214: 272
Ovarian hyperstimulation syndrome (OHSS)	An exaggerated systemic response to ovarian stimulation with a wide spectrum of clinical and laboratory manifestation. According to the degree of manifestations it is classified as mild, moderate, or severe.	Humaidan P, et al. Hum Reprod. 2016;31: 1997–2004
Venous thromboembolism (VTE)	Deep vein thrombosis (DVT), defined as a blood clot in deep veins of the legs with or without pulmonary embolism (PE). DVT is diagnosed if confirmed by Doppler ultrasound exam. PE diagnosis is confirmed by ventilation-perfusion lung scanning, angiography, or computed tomography. Pregnancy-related VTE is defined as a VTE occurring during the pregnancy or in the puerperium (within 6 weeks after delivery).	Kearon C, et al., Chest 2012; 141 (2 Suppl): e419S-e496S.
Hemorrhage.	Bleeding events. They are classified as major or minor according the ISTH criteria. Wound hematoma in case of caesarean section	Schulman S, J Thromb Haemost 2005; 3: 692–4.

### Data management

Data are stored and analysed in a central database. Access to the study documentation system and the study database requires user authentication and is restricted to the responsible study staff. Women are consecutively enrolled, so avoiding possible errors deriving from subject selection. Further errors deriving from a subjective interpretation of outcome measurements are avoided through using well-defined scores. Also, standardization of collection procedure has been thought to avoid errors due to data collection.

### Statistical analysis plan

Absolute numbers, percentage and median (range) or mean (±standard deviation) will be calculated to describe study groups and sub-groups. Associations between aforementioned primary and secondary outcomes and both numerical and categorical variables (age, drugs, co-morbidities, medical procedures, drug regimens and doses, etc.) collected during study will be tested. Relative risk value, with a 95% confidence interval, will be computed for the comparison of outcomes occurrence.

Validity of primary results will be examined, using a sensitivity analysis of sub-groups and applying more than one statistical test. Parametric and non-parametric statistical tests, according to data distributions, will be used for comparison of categorical and numerical variables. Strategy to prevent missing data will be employed. We select clinical information and laboratory measurements that are routinely included in the ‘real word’ practice, to facilitate the adherence of the investigators to the study protocol. Contacts will be kept with the investigators to reduce the burden of missing data as much as possible. A query-based system will allow us to ask investigators for the missing variables, whether the variables are meaningful for the final analysis. A *p* value below 0.05 will be considered to define statistical significance.

Stratification according to sub-groups of subjects will be taken into account on the basis of propensity to receive or not the treatment. This strategy will allow for an accurate and statistically valid analysis of heterogeneity that will be eventually observed. Potential confounding factors will be identified and after the evaluation of possible relationship between single variables by univariate analysis, logistic regression analysis will be performed to estimate the independence of associations identified. Data analysis will be performed also taking into account type of molecules administered, starting time and duration of thromboprohylaxis.

### Study size estimation

In the literature, the likelihood of observing live births without any treatment is estimated to range between 55 and 80% [2]; a multicentre Italian study [9] showed the occurrence of complications in 50% of all untreated women during pregnancy. Given these estimates, for the OTTILIA register, in order to observe an absolute increase of 15–20% (statistical power: 80%, significance level: 0.05), we calculated the inclusion of 190–340 thrombophilic women and, simultaneously, a cohort of pregnant women with the same clinical history (see inclusion criteria) not carrying inherited thrombophilia, in a ratio of at least 1:1. Thus, an overall number of 380–680 consecutive pregnancies are needed.

As far as the FIRST register is concerned, we estimated the inclusion of 624 women. This estimate is calculated on the basis of (1) the probability of observing live births after ART procedures (20–30%), (2) the estimate of an absolute increase of 5–10% in the number of live births after a prophylaxis of LMWHs (statistical power: 80%, significance level: 0.05).

It has been estimated that the OTTILIA and FIRST registries will be executed in the periods February 2012–December 2020 and October 2015–September 2020, respectively. In both the registries, an additional period of 9 months from the date of the last enrollment will be considered.

## Discussion

Recurrent reproductive failure represents a relevant women’s health problem. Despite increasing knowledge on this issue, the weight of some risk factors is still unclear and advancements in clinical management and treatment generate new unknown issues and uncertainties. Thus, in current clinical practice a lot of different approaches in management of spontaneous pregnancies or after ART attempts in women with previous failures are observed.

The OTTILIA and FIRST registries encompass population of pregnant women with a history of recurrent PL and women undergoing ART procedures after previous failures, respectively, to clarify the role of factors affecting outcome and identify strategies for a successful clinical management.

Although RCTs represent the ‘gold standard’ for evaluating treatment outcomes, studies in these populations are often interrupted before reaching sample size provided at the time of study design because of the slow recruitment and to the reluctance of women to participate, as previously anticipated. Due to their observational character, the OTTILIA and FIRST registries represent a valid alternative to RCTs, by systematically registering who is treated, how long, and which molecules are used. In addition, while RTCs are normally carried out under optimal conditions, very different from real life, observational studies provide a representative picture of the problem and show how treatments/interventions are administered in everyday clinical practice. Unlike previous observational studies, our registries encompass well-defined populations of women with recurrent reproductive failures; furthermore the multicenter approach allows to collect larger number of participants, different geographic locations, the possibility of inclusion of a wider range of population groups, and the ability to compare results among centers, all of which increase the generalizability of the study.

Thus, the strengths of these studies are primarily intrinsically associated with their characteristic design. Other strong points include the use of predefined questionnaires to ensure a standardised and comparable observation, and an electronic documentation system for high-quality data acquisition, in addition to a centralized data management. Last but not least, unlike usually happens in real life studies, in our registries the presence of eligibility criteria improve the consistency of the results, by enrolling women with specific characteristics. For example, in contrast to numerous clinical studies on recurrent PL, in the OTTILIA register, strict criteria are required for enrolment: ≥3 or 2 PL in the presence of at least 1 normal fetal karyotype.

Due to their proximity to the real world, with all its complexity, limitations of these registers include variable treatment adherence, multiple therapies, that often dilute the magnitude of a treatment effect or influence treatment decision. However, the choice of well-defined selection criteria should help prevent these risks.

Therefore, by remedying the problem of study design in obstetric populations, we believe that our registries will improve knowledge on mechanisms involved in reproductive failure and support clinical decisions in the prevention of this very serious and stressful women’s health problem.

## Data Availability

Not applicable.

## Abbreviations

ART: Assisted reproductive technologies
DVT: Deep vein thrombosis
ESHRE: European Society of Human Reproduction and Embryology
GH: Gestational hypertension
ICSI: Intracytoplasmic sperm injection
IUFD: Intrauterine fetal death
IUI: Intrauterine insemination
IVF: In vitro fertilization
LDH: Lactate dehydrogenase
LMWHs: Low-molecular-weight heparins
OHSS: Ovarian hyperstimulation syndrome
PE: Pulmonary embolism
PL: Pregnancy loss
RCTs: Randomized clinical trials
RPL: Recurrent pregnancy loss
SGA: Small-for-gestational age
VTE: Venous thromboembolism
